# Effectiveness of greenhouse gas mitigation intervention for health-care systems: a systematic review

**DOI:** 10.2471/BLT.23.290464

**Published:** 2023-01-31

**Authors:** Iris Martine Blom, Mohamed Eissa, Juliette Claudine Mattijsen, Hamaiyal Sana, Andy Haines, Sarah Whitmee

**Affiliations:** a Centre on Climate Change and Planetary Health, London School of Hygiene & Tropical Medicine, Keppel Street, Bloomsbury, London, WC1E 7HT, England.; b Alexandria Faculty of Medicine, Alexandria University, Alexandria, Egypt.; c Julius Centre for Health Sciences and Primary Care, UMC Utrecht, Utrecht, Kingdom of the Netherlands.; d Bolan Medical College, Quetta, Pakistan.

## Abstract

**Objective:**

To identify evidence-based interventions that reduce greenhouse gas emissions in health-care systems in low- and middle-income countries and explore potential synergies from these interventions that aid climate change adaptation while mitigating emissions.

**Methods:**

We systematically searched 11 electronic databases for articles published between 1990 and March 2023. We assessed risk of bias in each article and graded the quality of evidence across interventions in health-care operations, energy and supply chains.

**Findings:**

After screening 25 570 unique records, we included 22 studies published between 2000 and 2022 from 11 countries across six World Health Organization regions. Identified articles reported on interventions spanning six different sources of emissions, namely energy, waste, heating and cooling, operations and logistics, building design and anaesthetic gases; all of which demonstrated potential for significant greenhouse gas emission reductions, cost savings and positive health impacts. The overall quality of evidence is low because of wide variation in greenhouse gas emissions measuring and reporting.

**Conclusion:**

There are opportunities to reduce the greenhouse gas emissions from health-care systems in low- and middle-income countries, but gaps in evidence were identified across sources of emissions, such as the supply chain, as well as a lack of consideration of interactions with adaptation goals. As efforts to mitigate greenhouse gas intensify, rigorous monitoring, evaluation and reporting of these efforts are needed. Such actions will contribute to a strong evidence base that can inform policy-makers across contexts.

## Résumé

**Objectif:**

Identifier les interventions fondées sur des données factuelles qui visent à réduire les gaz à effet de serre émanant des systèmes de soins de santé dans les pays à revenu faible et intermédiaire, mais aussi explorer d'éventuelles synergies liées à ces interventions et susceptibles de contribuer à une meilleure adaptation au changement climatique tout en réduisant les émissions.

**Méthodes:**

Nous avons procédé à une analyse systématique de 11 bases de données électroniques, à la recherche d'articles publiés entre 1990 et mars 2023. Pour chaque article, nous avons déterminé le risque de biais et évalué la qualité des données relatives aux interventions dans les unités de soins de santé, l'énergie et les chaînes d'approvisionnement.

**Résultats:**

Après avoir examiné 25 570 documents uniques, nous avons conservé 22 études publiées entre 2000 et 2022, issues de 11 pays appartenant à six régions de l'Organisation mondiale de la Santé. Les articles retenus mentionnaient des interventions couvrant six sources d'émissions différentes, à savoir l'énergie, les déchets, le chauffage et la climatisation, les opérations et services logistiques, la conception des bâtiments, ainsi que les gaz anesthésiques; toutes ont révélé un potentiel en matière de réduction des émissions de gaz à effet de serre, de réalisation d'économies et d'impact positif sur la santé. Globalement, les données sont de piètre qualité en raison des variations des mesures et rapports concernant les émissions de gaz à effet de serre.

**Conclusion:**

Il existe plusieurs moyens de réduire les émissions de gaz à effet de serre provenant des systèmes de soins de santé dans les pays à revenu faible et intermédiaire. Cependant, les preuves sur les sources d'émissions manquent, notamment au niveau de la chaîne d'approvisionnement, et les interactions à des fins d'adaptation ne sont que rarement prises en compte. Alors que les efforts visant à réduire ces émissions se multiplient, il est impératif de les surveiller, de les évaluer et d'établir des rapports rigoureux. De telles actions contribueront à mieux étayer les données afin d'orienter les responsables politiques en fonction du contexte.

## Resumen

**Objetivo:**

Identificar intervenciones basadas en evidencias que reduzcan las emisiones de gases de efecto invernadero en los sistemas sanitarios de los países de ingresos bajos y medios y explorar las posibles sinergias de estas intervenciones que ayuden a adaptarse al cambio climático mientras se mitigan las emisiones.

**Métodos:**

Se realizaron búsquedas sistemáticas en 11 bases de datos electrónicas de artículos publicados entre 1990 y marzo de 2023. Se evaluó el riesgo de sesgo en cada artículo y se clasificó la calidad de las pruebas en las intervenciones relacionadas con las operaciones sanitarias, la energía y las cadenas de suministro.

**Resultados:**

Tras examinar 25 570 registros únicos, se incluyeron 22 estudios publicados entre 2000 y 2022 de 11 países de seis regiones de la Organización Mundial de la Salud. Los artículos identificados informaron sobre intervenciones que abarcaban seis fuentes diferentes de emisiones: energía, residuos, calefacción y refrigeración, operaciones y logística, diseño de edificios y gases anestésicos; todos ellos demostraron potencial para reducciones significativas de las emisiones de gases de efecto invernadero, ahorro de costes e impactos positivos en la salud. La calidad general de las evidencias es baja debido a la gran variación en la medición y la notificación de las emisiones de gases de efecto invernadero.

**Conclusión:**

Existen oportunidades para reducir las emisiones de gases de efecto invernadero de los sistemas de atención sanitaria en los países de ingresos bajos y medios, pero se han detectado deficiencias en las evidencias de las diferentes fuentes de emisiones, como la cadena de suministro, así como una falta de consideración de las interacciones con los objetivos de adaptación. A medida que se intensifican los esfuerzos para mitigar los gases de efecto invernadero, es necesario realizar un seguimiento, una evaluación y una notificación rigurosos de estos esfuerzos. Estas medidas contribuirán a crear una base de evidencias sólida que pueda informar a los responsables de formular las políticas en todos los contextos.

## ملخص

**الغرض:**

تحديد التدخلات القائمة على الأدلة، والتي تقلل من انبعاثات غازات الاحتباس الحراري في أنظمة الرعاية الصحية في الدول ذات الدخل المنخفض والدخل المتوسط، واستكشاف سبل الاندماج المحتملة من هذه التدخلات، والتي تساعد على التكيف مع تغير المناخ مع تخفيف الانبعاثات.

**الطريقة:**

قمنا بالبحث بشكل منهجي في 11 قاعدة بيانات إلكترونية للمقالات المنشورة من عام 1990 وحتى مارس/آذار 2023. وقمنا بتقييم خطر التحيز في كل مقالة، وتصنيف جودة الأدلة عبر التدخلات في عمليات الرعاية الصحية، والطاقة، وسلاسل التوريد.

**النتائج:**

بعد فحص 25570 سجلاً فريدًا، قمنا بتضمين 22 دراسة منشورة بين عامي 2000 و2022، من 11 دولة مختلفة عبر ست مناطق تابعة لمنظمة الصحة العالمية. إن المقالات التي تمت الإشارة إلى أنها تناولت التدخلات، تمتد عبر ستة مصادر مختلفة للانبعاثات، وهي الطاقة، والنفايات، والتدفئة والتبريد، والعمليات والخدمات اللوجستية، وتصميم المباني، وغازات التخدير؛ وقد أظهرت جميعها إمكانية تحقيق تخفيضات كبيرة في انبعاثات غازات الاحتباس الحراري، وتوفير التكاليف، وتحقيق تأثيرات إيجابية على الصحة. الجودة الإجمالية للأدلة منخفضة بسبب التباين الكبير في قياس انبعاثات غازات الاحتباس الحراري، والإبلاغ عنها.

**الاستنتاج:**

هناك فرص للحد من انبعاثات غازات الاحتباس الحراري من أنظمة الرعاية الصحية في الدول ذات الدخل المنخفض والدخل المتوسط، ولكن تم تحديد ثغرات في الأدلة في مصادر الانبعاثات؛ مثل سلسلة التوريد، فضلاً عن عدم مراعاة التفاعلات مع أهداف التكيف. ومع تكثيف الجهود الرامية إلى التخفيف من غازات الاحتباس الحراري، هناك حاجة إلى رصد هذه الجهود، وتقييمها، والإبلاغ عنها بشكل نشط. ستسهم مثل هذه الإجراءات في إنشاء قاعدة قوية للأدلة، يمكنها توعية واضعي السياسات في الأوضاع المختلفة.

## 摘要

**目的:**

确定可以减少低收入和中等收入国家卫生保健系统的温室气体排放的循证干预措施，并探索这些干预措施在支持减排的同时在帮助适应气候变化方面的潜在协同作用。

**方法:**

我们系统地检索了 11 个电子数据库，以查找 1990 年至 2023 年 3 月之间发表的相关文章。我们评估了每篇文章的偏倚风险，并对卫生保健业务、能源和供应链干预措施的证据质量进行了分级。

**结果:**

在筛选了 25,570 份不重复的记录后，我们纳入了 2000 年至 2022 年之间发表的 22 项研究，这些研究来自世界卫生组织六个区域的 11 个不同国家。已确定的文章报告了涉及六种不同排放源的干预措施，即能源、废物、供暖和制冷、运营和物流、建筑设计和麻醉气体；所有这些报告都表明干预措施有可能大幅减少温室气体的排放、节约成本并对健康产生积极的影响。由于温室气体排放测量和报告之间存在巨大差异，证据的总体质量较低**。**

**结论:**

在低收入和中等收入国家，减少卫生保健系统的温室气体排放是可能的，但不同排放源的证据质量之间存在差距（例如供应链），并缺乏对适应目标间相互影响的考虑。随着温室气体减排工作的不断加强，需要对这些工作进行严格的监测、评估和报告。这些举措将有助于构建强有力的证据基础，从而为不同背景下的政策制定者提供信息。

## Резюме

**Цель:**

Выявить научно обоснованные меры, направленные на сокращение выбросов парниковых газов в системах здравоохранения в странах с низким и средним уровнем дохода, и изучить возможный синергетический эффект от этих мер, способствующих адаптации к изменению климата и одновременно снижающих уровень выбросов.

**Методы:**

Авторы изучили 11 электронных баз данных в поисках статей, опубликованных в период с 1990 года по март 2023 года. Оценивался риск возникновения системной ошибки в каждой статье и качество доказательств по различным видам вмешательств в секторах работ в области здравоохранения, энергопотребления и цепочки поставок.

**Результаты:**

После проверки 25 570 уникальных записей в обзор были включены 22 исследования, опубликованные в период с 2000 по 2022 год в 11 разных странах, представляющих шесть регионов Всемирной организации здравоохранения. В найденных статьях сообщалось о мерах, охватывающих шесть различных источников выбросов, а именно: энергопотребление, отходы, обогрев и охлаждение, эксплуатацию и логистику, проектирование зданий и анестетические газы. Каждая из них продемонстрировала потенциал для значительного сокращения выбросов парниковых газов, экономии затрат и положительного воздействия на здоровье. Общее качество доказательств оценивается как низкое из-за большого разброса в измерении и отчетности по выбросам парниковых газов.

**Вывод:**

Существуют возможности для сокращения выбросов парниковых газов от систем здравоохранения в странах с низким и средним уровнем дохода, однако были выявлены пробелы в фактических данных по всем источникам выбросов, таким как цепочка поставок, а также недостаточное рассмотрение взаимодействия с целями адаптации. Поскольку предпринимаются все более активные усилия по сокращению выбросов парниковых газов, необходимы тщательный мониторинг и оценка этих усилий, а также отчетность о таких усилиях. Такие меры будут способствовать созданию прочной доказательной базы, которая будет служить источником информации для ответственных лиц в различных контекстах.

## Introduction

In the absence of actions to rapidly reduce global greenhouse gas emissions, climate change is predicted to be the biggest threat to human health in the 21st century. Direct and indirect health effects from climate change include exposure to extreme weather, undernutrition, the spread of vector-borne diseases, lack of access to clean water, and mental health effects.^1^ Health-care systems are facing the challenge of treating these impacts, but they also emit about 4.4% of global greenhouse gas emissions with projected increases in emissions.^2^^,^^3^ Since the United Nations Framework Convention on Climate Change 26th Conference of Parties in 2021 (UNFCCC COP26), 75(54 low- and middle-income) countries have committed to transitioning to sustainable, low-carbon health systems, with 29 (22 low- and middle-income) countries aiming to reach net-zero emissions in their health-care systems.^4^^,^^5^

Health-care systems in low- and middle-income countries emit lower per capita greenhouse gas emissions compared to those in high-income countries,^2^^,^^3^ but as health-care systems in many low- and middle-income countries advance, an increase in emissions is likely unless steps are taken to identify, measure and control them. Low- and middle-income countries are also predicted to experience the harmful effects of climate change with greater intensity and at an earlier stage due to their geographical location, exposure and vulnerability, while being less equipped to handle these effects due to a shortage of resources to cope and recover.^6^^,^^7^ Any adaptation actions undertaken by health-care systems should not exacerbate the health sector's greenhouse gas emissions, creating negative feedback loops and locking them into higher emission trajectories. 

To fulfil the commitments undertaken at, and since, COP26, it is necessary to identify evidence-based strategies for reducing the greenhouse gas emissions of health-care systems in low- and middle-income countries.^8^ We undertook a systematic review to identify modelled and implemented greenhouse gas mitigation interventions and their relationship with adaptation, applicable within the context of low- and middle-income countries, to provide evidence on which interventions are most feasible to implement and where actions can be scaled to provide significant reductions in emissions within health-care facilities and across the sector.

## Methods

We followed a protocol published on 4 August 2022^9^ following the Preferred reporting items for systematic review and meta-analysis protocols^10^ checklist (online repository).^11^ The protocol underwent one methodological amendment, namely the removal of the Joanna Briggs Institute Critical Appraisal Tools for evaluation, as they were not relevant to the types of interventions we analysed.^12^ We searched the database Ovid MEDLINE®, Ovid Embase®, Global Health, Web of Science, Africa-Wide Information, LILACS, Global Index Medicus, ELDIS, SCOPUS, AfricaPortal and GreenFILE on 17 March 2023. We predetermined the inclusion and exclusion criteria, which are detailed in Box 1.

Box 1 **Inclusion criteria for articles on greenhouse gas mitigation interventions for health-care systems**

**Publication types**

Peer-reviewed primary research including analytical cross-sectional studies, case-control studies, case reports, cohort studies, diagnostic test accuracy studies, and randomized controlled trials. We excluded other types of publications, such as protocols, guidelines, (systematic) reviews, perspectives, commentaries or editorials. We screened relevant reviews for primary research references.

**Languages**

No restriction.

**Context**

Findings of research in one or more low- and middle-income countries.

**Topic**

Any implemented or modelled greenhouse gas mitigation intervention across health-care operations, energy and supply chains.

**Outcome**

Reporting a quantified change in greenhouse gas emissions from the intervention.

**Timeline**

Published between 1990 and 17 March 2023. Year 1990 was chosen as a starting point for the inclusion of articles, as a significant number of publications supporting a connection between climate change and health started to appear in the early 1990s.^13^^,^^14^

### Search strategy

Our search strategy consisted of three main elements: (i) the health-care system; (ii) greenhouse gases; and (iii) low- and middle-income countries (Box 2 and online repository).^9^^,^^15^ To further structure our strategy, we devised a conceptual theory of change framework. We used approaches outlined by the United Nations Sustainable Development Group Latin America and the Caribbean and the New Philanthropy Capital and insights from a previous publication to develop this framework.^16^^,^^17^ The framework is defined in (Box 3; available at: https://www.who.int/publications/journals/bulletin/) and detailed descriptions of each section can be found in our online repository.^15^

Box 2 **Search strategy, search line and content of search parameters to identify articles on greenhouse gas mitigation interventions for health-care systems**

1: (netzero or net zero).mp.

2: carbon footprint/

3: greenhouse effect/

4: exp climate change/

5: (carbon or CO2 or methane or CH4 or nitrous oxide or N2O or hydrofluorocarbon* or HFC* or perfluorocarbon* or PFC* or F-gas or fluorinated gas or sulfur hexafluoride or SF6 or nitrogen trifluoride or NF3 or emission* or greenhouse or GHG or climate change* or global warming or footprint or eco-friendly or climate friendly or environment* friendly or eco-efficient or environment* responsible or environment* sound or energy-efficient or energy-saving or green initiative* or environmental impact or short-lived climate pollutant or black carbon).mp.

6: (environment* and sustainable*).mp.

7: 1 or 2 or 3 or 4 or 5 or 6

8: exp “delivery of healthcare”/

9: exp health facilities/

10: (health system* or health care or healthcare or health sector or health supply chain* or health service* or delivery of health or health delivery or health facility* or health cent* or hospital or hospitals or clinic or clinics or emergency department* or operating* room* or operating* theatre* or patient care or ward* or urgent care or primary care or secondary care or tertiary care or quaternary care or telemedicine or medical cent* or diagnostic care or rehabilitative care or preventative care or palliative care or home care).mp.

11: 8 or 9 or 10

12: 7 and 11

304: or/13–303 [ALL LOW AND MIDDLE-INCOME COUNTRIES (expert search)]

305: 12 and 304

306: limit 305 to yr = ”1990–2023”

Box 3 **Conceptual framework according to the theory of change on greenhouse gas mitigation interventions in health-care systems in low-and middle-income countries**

***Problem statement***

Climate change is and will continue to affect human health through many different direct and indirect health outcomes. Less well-known is that health-care systems themselves contribute 4.4 % of global greenhouse gas emissions. Health-care systems, referring to the institutions, people and resources involved in delivering health care to individuals, need to implement mitigation interventions to ensure an adequate, effective and systematic response to these health effects while aiming for synergies or co-benefits with adaptation and, specifically, climate resilience. Since UNFCCC COP26, countries have committed to a more environmentally sustainable, low-carbon health-care system – out of which the majority are low- and middle-income countries. There is a lack of robust evidence guiding efforts towards environmentally sustainable health-care systems, particularly in low- and middle-income countries.

***Impact and aim***

If measures are taken to mitigate greenhouse gas emissions produced by health-care systems in low- and middle-income countries effectively, then:

1. the health-care systems could advance while contributing less to climate change;

2. a knock-on effect could potentially lead to a reduction in climate risk for health due to synergies or co-benefits for adaptation; and

3. raising awareness can indirectly help achieve local and national climate goals. This happens as people, communities, and other sectors, including high-income countries, become more informed about how climate change affects health. This knowledge can lead to better climate actions as well as improving climate plans by combining them with health strategies. Furthermore, the health-care sector can significantly guide and shape the actions of these various groups.

*Delivery assumptions:*

1. Relevant interventions can be identified in the literature

2. Sufficient interest and dedication from policy-makers

3. Skills, abilities and resources are present.

*Assumptions about effects:*

1. Improved health outcomes through interventions

2. Potential positive knock-on effect on adaptation

3. Potential indirect effect on awareness and local and national climate action.

*Possible unintended consequences*

1. Conflict or trade-off mitigation intervention with adaptation or prioritization mitigation over adaptation when there is an urgent need to adapt.

*Theory of change process assumptions*

1. Robust data and experts consulted

2. Theory of change is a living document.

***Outcomes, outputs and potential risk and barriers***

1. Reduction of greenhouse gas emissions produced by health-care operations (emission scope 1).

Key Indicator: percent reduction in greenhouse gas emissions.

- Stimulate low carbon prescriptions
- Increase efficiency and minimize patient travel, that is, through strategic planning and multidisciplinary consults
- Transition to a health-care system of community-based health promotion and disease prevention with a prominent role of primary health care
- Shift towards higher usage of eHealth, including teleconsultations
- Stimulate the use of low carbon transport alternatives for operations, including low emission ambulances
- Health workforce barriers including lack of adequately trained health workers might prevent multidisciplinary consults, a transition to preventive, primary health care
- Lack of access to technology might prevent eHealth
- Soft issues such as lack of support and awareness among staff, open dialogue and proper infrastructure to implement change.

Note: Financial barriers or other accessibility barriers including patents might prevent low-carbon prescriptions or low-carbon transport alternatives.

2. Reduction of greenhouse gas emissions from energy used in health care (emission scope 2).

Key Indicator: Percent of reduction in greenhouse gas emissions.

- Transition to clean energy through renewable energy sources and low carbon grids
- Use of batteries to expand the renewable energy supply
- Use energy efficiently, such as light-emitting diode (LED) fixtures
- Soft issues, including lack of support and awareness among staff or suppliers, lack of open dialogue, and lack of proper infrastructure to implement change.

Note: Financial barriers or other accessibility barriers including lack of expertise might prevent a transition to clean and renewable energy, use of battery power energy efficient products such as LED lighting.

3. Reduction of greenhouse gas emissions of health-care supply chains (emission scope 3).

Key Indicator: Percent of reduction in greenhouse gas emissions.

- Reuse of medical devices and supplies
- Reduce the acquisition of non-reusables and high-emission alternatives and increase the use of low-emission alternatives
- Transition to a predominantly plant-based hospital menu with locally-produced foods (e.g. for staff and visitors)
- Stimulate health and care workers and patients to minimize transport and, when necessary, use active transport or electric, shared vehicles
- Use low-emission alternatives for transportation and distribution
- Encourage low-emission travel options for business travels
- Procure from net-zero suppliers or suppliers with a strategy to move to net-zero
- Food system effects or food availability might prevent a transition to plant-based hospital menus with locally-produced food
- Soft issues, including lack of support and awareness among staff or suppliers, lack of open dialogue, and lack of proper infrastructure to implement change.

Note: Financial barriers or technological limitations might prevent reuse of supplies, low-emission prioritization in acquisitions, low-emission alternatives for transportation or distribution, low-emission travel options, and procuring from net-zero suppliers.

4. Co-benefit or synergy of the mitigation intervention with actions contributing to climate change adaptation.

Key Indicator: Percent of reduction in loss of life or disability.

- Hospital-wide passive heating and cooling system
- Agriculture on hospital rooftops
- Soft issues, including lack of support and awareness among staff and/or leadership, lack of open dialogue, and lack of proper infrastructure to implement change.

Note: Financial barriers due to specified or allocated funding, lack of flexibility of funding and gaps in knowledge.

COP: Conference of Parties; LED: light-emitting diode; UNFCC: United Nations Framework Convention on Climate Change.Note: Adapted from Rasheed et al., 2021.^17^

### Selection process and data extraction

We uploaded records using Rayyan QCRI software (Rayyan, Cambridge, United States of America), and the aforementioned inclusion and exclusion criteria were applied throughout the screening process. Following published efficiency guidelines,^18^ we removed duplicates, screened titles and analysed abstracts and full texts against eligibility criteria using Rayyan QCRI. Two reviewers performed each step separately, after which any disagreements were discussed. If no consensus was reached, a third author was consulted for resolution. Two reviewers independently extracted all relevant data from eligible articles using a pre-tested form with detailed instructions (Box 4). This extracted data was used to generate a 100-word or less summary on the extraction sheet.

Box 4 **Data extracted for each article identified in the systematic review on greenhouse gas mitigation interventions for health-care systems**

***Article identifiers:***

Basic identifiers including name, authors, date, journal, article type and article design

***Methods:***

Types of research methods used in the article

***Geographical scale:***

Whether the study was conducted at a local, regional, national or international level

***Location:***

Relevant town or city, region, country and/or countries where the research was conducted

***Emission scope:***

Health-care operations (scope 1), energy (scope 2), supply chains (scope 3)

***Part of the health-care system:***

A particular aspect of the health-care system such as a primary health-care facility or a rural hospital

***Greenhouse gas mitigation intervention(s):***

Intervention details that lead to a decrease in greenhouse gas emissions

***Measurable effects of the greenhouse gas mitigation intervention(s):***

Quantified effects of the identified intervention(s) on mitigation, including a specification of greenhouse gas or carbon dioxide equivalent and whether it was measured or modelled

***Implementation process:***

A description of the implementation process, including enablers and barriers and how these were approached

***Implementation timeline:***

Timeline of the implementation process

***Economic analysis:***

Any provided economic information such as cost–effectiveness, cost–benefit or cost consequences

***Linkage with adaptation or resilience:***

Whether the intervention was directed at both mitigation and adaptation or if resilience was described. These interactions can be synergies, co-benefits, conflicts, trade-offs or co-harms^19^

***Health effects:***

Measured effects on health outcomes or exposures

***Funding source:***

Source of funding for the authors

***Conflicts of interest:***

Further potential conflicts of interest, including relationships with relevant parties other than financial relationships

We assessed risk of bias using specifically designed questions intended to be applicable across different study types using a simple judgement of low risk, high risk or unclear risk on different axes as endorsed by the Cochrane Collaboration.^42^ Independent assessments were made by at least two authors. 

We assessed the overall strength of evidence resulting from article synthesis using the Grading of recommendations assessment, development, and evaluation (GRADE) approach. The collated evidence was graded using four different categories: (i) very low (we believe the true effect is probably very different from the estimated effect); (ii) low (we believe the true effect might be very different from the estimated effect); (iii) moderate (we believe that the true effect is probably close to the estimated effect); or (iv) high (we are confident that the true effect is similar to the estimated effect).^43^ We used GRADEpro Guideline Development Tool (McMaster University and Evidence Prime, Hamilton, Canada) for the analysis.

## Results

**Fig. 1:**
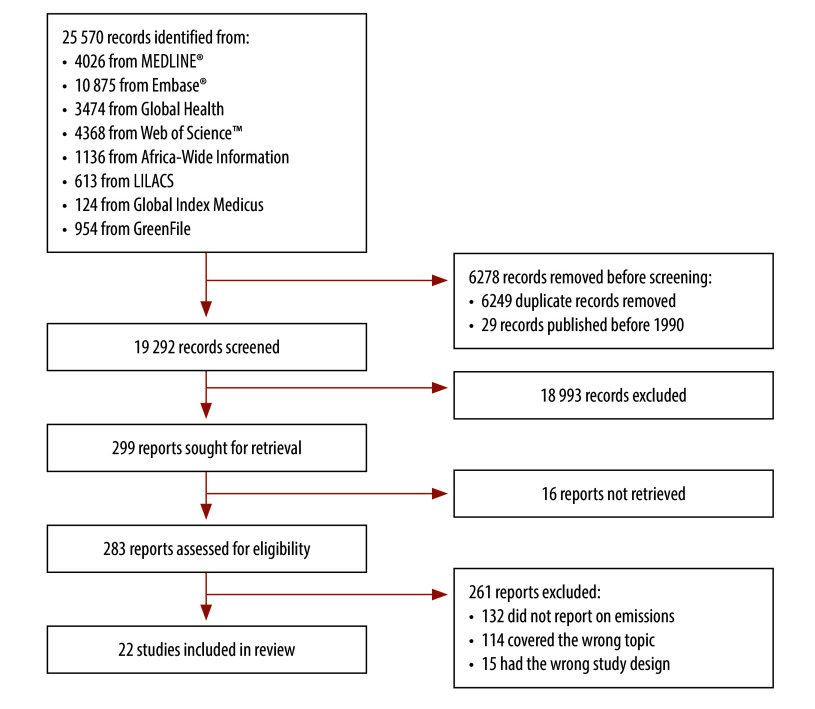
Flowchart of the selection of studies on greenhouse gas mitigation interventions for health-care systems

**Fig. 2:**
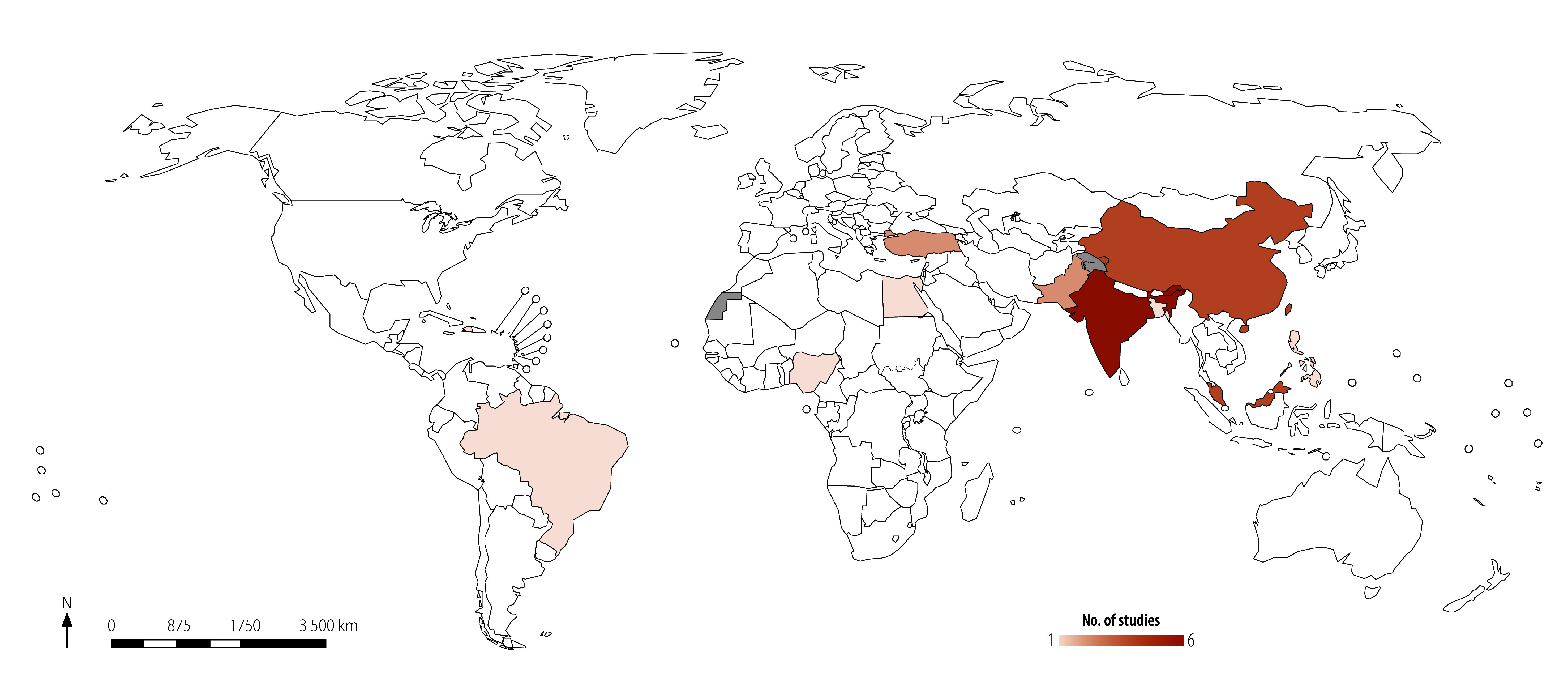
Geographical distribution of the included studies on greenhouse gas mitigation interventions for health-care systems

Our search yielded 25 570 records. After removing duplicates and screening the titles, abstracts and full texts, 22 articles met the inclusion criteria (Fig. 1).^20^^–^^41^ The 22 studies were published between 2000 and 2022, with 77% (17) of studies published between 2016 and 2022, and 36% (eight studies) between 2020 and 2022. They cover 11 countries across all World Health Organization (WHO) regions, primarily in the Western Pacific Region (seven studies) and South-East Asia Region (seven studies). India is the most-reported country (six studies; Fig. 2). Countries range from lower- to upper-middle-income countries, as per World Bank classification, with no low-income countries represented.^44^ Study settings vary from regional systems to urban areas, hospitals and rural centres (Table 1).

**Table 1:** Detailed summary of included studies on greenhouse gas mitigation interventions for health-care systems

Study	Study design	Year of intervention	Country, WHO region	Income level	Health system level	Study site(s)
Ahmadzadehtalatapeh & Yau, 2011^38^	Analytical and modelling	NR	Malaysia, Western Pacific Region	Upper-middle-income	Hospital ward	One orthopaedic ward
Ali et al., 2016^30^	Descriptive: cross-sectional	2014–2015	Pakistan, Eastern Mediterranean Region	Lower-middle-income	Hospital	Tertiary hospital
Chowdhury et al., 2021^20^	Descriptive: case report	NR	Bangladesh, South-East Asia Region	Lower-middle-income	Health-care facility	One temporary rural health-care centre on an island
Ciplak, 2015^31^	Descriptive: cross-sectional	NR	Türkiye, European Region	Upper-middle-income	Region within country	One region
Datta et al., 2016^39^	Analytical: experimental	2015	India, South-East Asia Region	Lower-middle-income	Outpatient surgery	Paediatric eye examinations at one hospital
Duraivelu & Elumalai, 2021^21^	Descriptive: case report	2019	India, South-East Asia Region	Lower-middle-income	Hospital	One urban hospital
Isa et al., 2016^22^	Analytical and modelling	NR	Malaysia, Western Pacific Region	Upper-middle-income	Hospital	One university hospital
Khan et al., 2019^32^	Descriptive: case series	2016–2017	Pakistan, Eastern Mediterranean Region	Lower-middle-income	Clinic	371 private clinics
Khor et al., 2020^33^	Analytical: observational: case-control	2017	Malaysia, Western Pacific Region	Upper-middle-income	Hospital	One hospital
Lemence & Tamayao, 2021^23^	Analytical and modelling	NR	Philippines, Western Pacific Region	Lower-middle-income	Health-care facility	One rural health-care facility
Liu et al., 2022^34^	Analytical and modelling	2050	China, Western Pacific Region	Upper-middle- income	Health-care system	Hospitals, community health service centres, township health centres, and village clinics
Narang et al., 2017^24^	Descriptive: case report	2015–2016	India, South-East Asia Region	Lower-middle-income	Clinical laboratory	One laboratory
Olatomiwa et al., 2018^25^	Descriptive: case series	NR	Nigeria, African Region	Lower-middle-income	Clinic	Six rural clinics in six different regions
Paksoy et al., 2000^26^	Descriptive: case report	NR	Türkiye, European Region	Upper-middle-income	Hospital	One university hospital
Panwar et al., 2013^27^	Analytical and modelling	2011–2012	India, South-East Asia Region	Lower-middle-income	Health-care system (subnational)	One city
Pina et al., 2021^28^	Analytical and modelling	NR	Brazil, Region of the Americas	Upper-middle-income	Hospital	One university hospital
Raghuwanshi & Arya, 2020^29^	Descriptive: case report	NR	India, South-East Asia Region	Lower-middle-income	Health-care facility	One remote health-care centre
Raila & Anderson, 2017^35^	Analytical: experimental	2014	Haiti, Region of the Americas	Lower-middle-income	Health-care system (subnational)	Five health-care waste incinerators
Sun & Huang, 2017^40^	Analytical and modelling	NR	China, Western Pacific Region	Upper-middle-income	Outpatient surgery	Lobby of outpatient department of a hospital
Thiel et al., 2017^41^	Descriptive: case series	2014	India, South-East Asia Region	Lower-middle-income	Surgery	2 tertiary care centres
Zakaria et al., 2005^36^	Descriptive: cross-sectional	NR	Egypt, Eastern Mediterranean Region	Lower-middle-income	Health-care system (subnational)	Six hospital waste incinerators
Zhao et al., 2021^37^	Analytical and modelling	NR	China, Western Pacific Region	Upper-middle-income	Health-care system (subnational)	One city

NR: not reported; WHO: World Health Organization.

Note: Income level follows the classification of the World Bank.^44^

### Interventions

Of the selected articles, we identified six primary intervention areas: energy (10 studies); waste (eight studies); heating and cooling (one study); operations and logistics (one study);building design (one study); and anaesthetic gases (one study). All articles detailed implementation; 14 discussed costs; 13 reported health effects; and one considered adaptation to the effects of climate change.

Twenty articles included data on carbon dioxide reduction whereas only two articles reported on other greenhouse gases or pollutants (Table 2). For one article, we could only extract percent reduction of emissions^20^ and for five others no percentage could be calculated as original emissions were not provided.^21^^,^^26^^,^^31^^,^^33^^,^^40^ Three articles^24^^,^^38^^,^^40^ only reported decreases in electricity usage, which was converted to carbon dioxide equivalent using the national grid emission factor.^45^^,^^46^ Two articles^24^^,^^27^ included a 100% reduction of carbon dioxide emissions and in this case the supply chain, installation of the system and relevant upkeep were not considered. Three articles indicated more than 100% reduction due to zero-emission electricity generation and selling the surplus.^28^^,^^32^^,^^38^ The intervention areas of energy and waste are outlined below, and the other four areas are described in Box 5.

**Table 2:** Interventions and outcomes in studies on greenhouse gas mitigation interventions for health-care systems

Country, reference	Scope and intervention type	Summary of intervention	Type of outcome measurement	Reduction CO_2_(equivalent) kg/year unless otherwise stated (%)	Reduction of other greenhouse gases per year unless otherwise stated
Bangladesh^20^	Electricity: Energy	A hybrid photovoltaic-converter-wind-battery-generator energy generation system for a temporary health centre is compared to: System A: a hybrid wind-generator-converter-battery system; and System B: a hybrid photovoltaic generator-converter-battery system	Modelled	Compared to: System A: NR (27) System B: NR (25)	Compared to system A: CO: 20 496 kg PM: 124 kg Unburned hydrocarbon: 895 kg SO_2_: 6 569 kg^b^ NOx: 19 254 kg
India^21^	Electricity: Energy	A 5-kWp on-grid solar photovoltaic rooftop system for one urban hospital is compared to solely grid-provided electricity	Modelled	11 287 (NR)	SO_2_: 8.86 kg^b^ NOx: 18.50 kg Ash: 485.792 kg
Malaysia^22^	Electricity: energy and heating	A grid-connected photovoltaic-fuel cell-battery system for energy and heating of one university hospital building is compared to a standard, standalone diesel system	Modelled	71 004 (74)	CO: 239 kg Unburned hydrocarbon: 26.4 kg PM: 18 kg SOx: 83 kg NOx: 2075.5 kg
Philippines^23^	Electricity: energy	A solar photovoltaic panel energy system with and without grid connection for a rural health-care facility is compared to a grid-only system	Empirical	With: 19 598 (59) Without: 62 776 (72)	NR
India^24^	Electricity: energy	A solar photovoltaic panel for a laboratory is compared to electricity from the grid	Modelled	13 860 (100)^a^	NR
Nigeria^25^	Electricity: energy	Optimal hybrid renewable system configurations for electricity generation (photovoltaic-wind-diesel-battery hybrid system configuration and photovoltaic-diesel-battery hybrid system configuration depending on the location) for six rural clinics from six different areas are compared to a diesel generator system	Modelled	20 113 (83)	NR
Türkiye^26^	Electricity: energy, heating and cooling	Using solar energy in combination with aquifer thermal energy storage for electricity generation for heating and cooling for one university hospital is compared to using oil and the electricity grid	Modelled	2 100 000	SOx: 7 000 kg NOx: 8 000 t
India^27^	Electricity: energy	A solar photovoltaic tunnel dryer for surgical cotton for one city is compared to a dryer on: light diesel oil or liquefied petroleum gas	Modelled	Compared to: Diesel: 12 150 (100) Gas: 6 720 (100)	NR
Brazil^28^	Electricity: energy	A hybrid polygeneration system for the provision of electricity to a hospital under four legal scenarios is compared to standard usage of the electricity grid. The legal scenarios are: 39.1: Purchase only: no sale of electricity allowed; 39.2: Annual consumer: purchase and sale are allowed with the condition of purchasing more electricity than sales annually; 39.3: Unrestricted sale: purchase and sale are allowed with no restraints; and 39.4: Excess electricity production is injected into the distribution network, creating energy credits in kWh, by means of a free loan.	Modelled	39.1: 4 852 036 (63) 39.2: 6 844 207 (90) 39.3: 17 774 491 (233) 39.4: 17 774 491 (233)	NR
India^29^	Electricity: energy	A photovoltaic-diesel-battery energy system for energy generation for a remote health-care centre is compared to a diesel-battery energy system	Modelled	1813 (46)	CO: 4.48 kg Unburned hydrocarbons: 0.496 kg PM: 0.337 kg SO_2_: 3.64 kg^b^ NO: 40 kg
Pakistan^30^	Supply chain: waste	An integrated system of hospital solid waste treatment and disposal consisting of composting, incineration, and material recycling is compared to the standard scenario of incineration and landfill or incineration only	Empirical	Compared to: Standard: 2 806 (62) Incineration only: 2 610 (47)	NR
Türkiye^31^	Supply chain: waste	A regional health-care waste management scenario of a centralized autoclave coupled with an incinerator is compared to: Scenario 1: an incinerator; Scenario 2: decentralized autoclaving coupled with an incinerator	Modelled	Compared to: Scenario1: 1 544 000 Scenario 2: 1 767 000	NR
Pakistan^32^	Supply chain: waste	Segregation into medical waste (which is incinerated with transportation by motorbikes and then sent to landfill), and general waste (from which material is recovered or composted and then sent to landfill), is compared to: Scenario 1: segregation with landfilling of general waste and incineration of medical waste, then landfilling, and Scenario 2: incineration and then landfilling of all waste	Empirical	Compared to: Scenario 1: 538 per tonne of waste (114) Scenario 2: 1 110 per tonne of waste (106)	NR
Malaysia^33^	Supply chain: waste	Segregation and recycling of waste of phacoemulsification surgery is compared to no segregation and recycling in one hospital	Empirical	0.139 per case	NR
China^34^	Supply chain: waste	Plastic recycling in the health-care system is compared to no recycling	Modelled	868 700 000 (57)	NR
Haiti^35^	Supply chain: waste	Mainstreaming the use of cardboard sharps health-care waste containers instead of plastic containers at five health-care waste incinerators	Empirical	NR	Black carbon: 61.68%
Egypt^36^	Supply chain: waste	Comparing a newer incinerator including a high-performance scrubber control system and good practice processes by an experienced operator, with an older incinerator without specified processes	Empirical	NR	CO: 3 358 mg/m^3^ (86.8)
China^37^	Supply chain: waste	Medical waste management in a city through microwave sterilization with landfill medical waste disposal technology is compared to: rotary kiln incineration; pyrolysis incineration; plasma melting and steam sterilization with landfill	Modelled	Compared to: Per disposal rotary kiln: 285 (68) Pyrolysis: 52 (28) Plasma melting: 551 (80) Steam sterilization: 30 (18)	NR
Malaysia^38^	Electricity: heating and cooling	An eight-row pipe heat exchanger system added to the air conditioning system in one orthopaedic ward in a university hospital is compared to a standard air conditioning system	Modelled	314 (147)^b^	NR
India^39^	Health-care operations: anaesthetic gases	Induction dose only sevoflurane during paediatric eye examination for children aged 1–5 years at one hospital is compared to standard low-flow sevoflurane	Empirical	7700 (22) per day of 10–12 procedures	CO_2_ equivalent includes a reduction of N_2_O of 3.75 L/case
China^40^	Electricity; building design	The energy consumption of an outpatient hospital lobby building design of a lobby of 16 m^2^ with two exterior walls, south-oriented at the same height as the rest of the hospital is compared to lobby designs that have a different number of exterior walls, a different orientation, and a different height. Then, different window–wall ratios and skylight ratios are compared	Modelled	186–1011^a^	NR
India^41^	Health-care operations, electricity and supply chain: operations and logistics	Usage of multiuse vial for pharmaceuticals, a short surgical duration, and a quick turnaround time during cataract surgery is compared to the standard practice in a British hospital	Empirical	124 (95) per case	NR

CO: carbon monoxide; CO_2_: carbon dioxide; kWp: kilowatt peak; N_2_O: nitrous oxide; NOx: nitrogen oxides; NR: not reported; PM: particulate matter; SO_2_: sulfur dioxide; SOx: sulfur oxides.

^a^ Emissions calculated using national emission factors.^45^^,^^46^

^b^ SO_2_ is a cooling aerosol, so reduced SO_2_ emissions partly offset the reduction of the heating effect from mitigation of greenhouse gas emissions.

Box 5 **Other greenhouse gas mitigation interventions in health-care systems**

***Heat exchanger system, Malaysia***

A hospital ward in Malaysia incorporated an eight-row heat pipe heat exchanger into its air conditioning system, yielding savings equivalent to approximately 314 kg of carbon dioxide each year. This system also provides an economic benefit of about US$ 42 000 annually with a payback period of 1.6 years, and offers the added advantage of preventing *Legionella* growth in the ducting system.^38^

***Sevoflurane use, India***

Using only the induction dose of sevoflurane for brief paediatric eye examinations in children aged 1–5 years reduced emissions in comparison to the traditional continuous low flow. Despite the high global warming potential of sevoflurane, this reduction in usage amounts to a modest climate benefit and cost savings of US$ 10 per day across 8–12 patients, enhancing health equity and affordability of this vital anaesthetic for children in low-resource settings.^39^

***Building design, China***

A hospital's new outpatient lobby design in a colder region of China, featuring two south-facing exterior walls over a 16 m^2^ area, is expected to achieve a significant reduction in carbon dioxide emissions, between 186 and 1011 kg annually, due to the decreased need for heating.^40^

***Multiuse pharmaceuticals and reusing surgical supplies, India***

Cataract surgery at the Aravind Eye Care Centre in India, when compared with similar procedures in the United Kingdom of Great Britain and Northern Ireland, showed that implementing multiuse pharmaceuticals and reusing surgical supplies led to a substantial 95% relative reduction in emissions. The centre also optimized surgical duration and turnaround times, running two adjacent operating rooms simultaneously, which contributed to better patient outcomes and lower complication rates. Nonetheless, the assessment acknowledged methodological limitations, including variance in greenhouse gas measurement techniques and a lack of life cycle inventories specific to India. The researchers advocated for the expansion of such interventions, suggesting new vision centres and the integration of telemedicine, supported by rigorous training and strict sterilization protocols. They highlighted that policy changes, particularly those allowing multiuse pharmaceuticals in more countries, are essential to mitigate the environmental impact of health-care practices.^41^

US$: United States dollars.

#### Energy interventions

We identified reports on hybrid energy systems using a combination of non-renewable and renewable energy sources^20^^–^^22^^,^^25^^,^^26^^,^^28^^,^^29^ or fully renewable sources;^23^^,^^24^^,^^27^ achieving carbon dioxide emission reductions of 25%–233% as compared to alternative scenarios (Table 2) where the reductions higher than 100% are attributed to surplus electricity generation exported to the grid. All reported energy systems featured solar photovoltaic electricity generation paired with various other sources, such as wind or diesel. Greenhouse gas emissions from production and installation were generally not considered, and no unintended consequences were reported. One article compared legal contexts and concluded that flexibility to sell or export electricity to the grid maximizes annual carbon dioxide emission savings.^28^

##### Implementation

We found that all study authors recognized hybrid energy systems as acceptable interventions when considering various factors such as electricity generation, environmental impact and economic feasibility. Photovoltaic electricity generation was also found to be environmentally, technically and economically feasible.^20^^–^^22^^,^^28^

The authors of two studies noted that these energy forms are scalable in rural health-care facilities in disparate geographical locations provided that local energy costs and climate parameters are considered during the pre-planning stages.^20^^,^^23^^–^^25^^,^^28^Scalability could extend to commercial buildings and agricultural industries as well.^21^^,^^27^

Initial capital costs and access to sufficient finance may act as a barrier to implementation of hybrid energy systems, but hybrid energy systems were seen as a solution to enhance energy reliability and reduce energy costs over time.^23^ Suggested solutions included government funding, international climate-related financing and renewable energy purpose obligations; with one article suggesting a 25-year implementation period.^21^^–^^23^ Wind and solar potential significantly influences their implementation, as areas with high potential (for example, those with strong insolation for solar energy), are more conducive to successful deployment than low-potential areas.

##### Economic analysis

Eight articles reported details on costing, including their Net Present Costs (ranging from 3658 to 146 284 United States dollars, US$), payback periods (ranging from 3.38 to 9.9 years), and return metrics, which vary across different systems and locations (Table 3).

**Table 3:** Studies reporting economic outcomes for greenhouse gas mitigation interventions for health-care systems

Country	Intervention	Initial capital, US$	Net present cost, US$	Payback period, year	Return on investment, %	Initial rate of return, %
Bangladesh^20^	Photovoltaic Converter-Wind-Battery-Generator energy generation system	NR	69 377 300	7	NR	NR
India^24^	Solar panel	12 000	NR	NR	NR	NR
India^27^	Solar photovoltaic tunnel dryer for surgical cotton	NR	10 660	3.38	86 to 150	NR
India^29^	Photovoltaic-diesel-battery energy system	NR	13 523	9.9	NR	NR
India^21^	5-kWp on-grid solar photovoltaic rooftop system	3 658	NR	7.1	NR	NR
Malaysia^22^	Grid-connected photovoltaic fuel cell-battery system	NR	98 318	NR	NR	NR
Nigeria^25^	Optimal hybrid renewable system configurations for electricity generation	NR	71 210 to 108 920	NR	NR	NR
Philippines^23^	A solar photovoltaic panel energy system with or without grid connection	NR	With: 87 139 Without: 146 284	With: 9.7 Without: 4.5	With: 6.10; Without: 15.90	With: 9.0 Without: 20.8

kWp: kilowatt peak; US$: United States dollars.

##### Health and health equity

Five articles qualitatively estimated potential health effects, noting that reliable hybrid energy systems can prevent power interruptions and address the lack of access to reliable electricity in rural areas. Without continuous access to electricity, the lack of essential medical equipment – such as incubators, ventilators and basic lighting, critical for safe childbirth and neonatal care – leads to a high rate of maternal and perinatal mortality; spoilage of medication; and the inability to sterilize medical equipment used in operating rooms. In addition to the negative effects noted above, lack of coordination and communication (hindered by lack of reliable access to electricity or broadband wireless networks) was also found to disproportionately affect the health care of women and children. Reliable electricity access can reduce these effects by increasing operating hours, attracting a larger health workforce, improving cold-chain for vaccines and medicines, and enhancing communication among health workers and between patients and health workers.^20^^,^^23^^–^^25^

Other important actions such as replacing diesel generators with hybrid systems can act to reduce harmful exposure to pollutants including unburned hydrocarbons and particulate matter; potentially reducing risks for lung cancer, asthma and bronchitis;^29^ as well as contributing to a safer work environment particularly in laboratory settings.^24^

##### Adaptation

Authors of one study examined the intersection of mitigation and adaptation in the context of a solar photovoltaic energy system with and without grid-connection for a rural health-care facility in the Philippines. They defined a climate-resilient energy system as providing “reliable, safe, and secure electricity during short‐term disasters and events and as longer‐term climate changes occur”, and found that this solar photovoltaic energy system could enable continued provision of care during both short- and longer-term climate change effects.^23^^,^^47^

#### Waste interventions

Of the eight studies on waste that we identified, one study covered plasma melting; used for melting medical waste. Plasma melting appears to have the highest overall relative greenhouse gas emissions as compared to alternative waste interventions.^37^ Four studies covered stand-alone incineration and a mix of incineration with landfilling or autoclaving, which have the second highest emission.^30^^–^^32^^,^^37^ Relative emission reductions can be achieved by centralizing the autoclave, ensuring efficient transportation and having well-trained operators.^31^^,^^36^ One article also considered water usage, and found that combining autoclaving with incineration may conserve 38 967 m^3^ of water annually compared to incineration alone (Table 2).^31^

Systems integrating waste segregation, composting and material recycling, all while optimizing transport, achieved the greatest emission reductions, ranging from 47%–114%.^30^^,^^32^^–^^34^ Any further reductions in emissions were achieved through material recovery.^32^ For example, cardboard sharps containers were found to reduce black carbon emissions by 62% compared to plastic sharps containers in an incineration-only system.^35^

Reported methodological limitations around waste management data include: (i) neglecting heat recovery;^30^^,^^37^ (ii) lack of accurate waste data;^32^ (iii) inability to measure electricity during operations and autoclaving;^33^ (iv) foreign emission factors;^33^ and (v) omission of transportation.^34^^,^^37^ Unintended negative consequences of waste management include ineffective segregation leading to exposure to hazardous items,^30^ and generation of toxic dioxin during recycling.^34^

##### Implementation

Appropriate waste management also acts to improve health and safety while reducing greenhouse gas emissions.^32^ Three articles recommended scaling up the proposed waste management systems within their respective cities and regions,^30^^–^^32^ one more broadly across low- and middle-income countries,^31^ while another recommended a global ban on plastic sharps containers.^35^ For example, composting of biodegradable waste in Pakistan was easy to implement because of low management and operation costs.^32^ In Türkiye, incineration on its own was not feasible due to high costs.^31^ Ultimately, widespread segregation and material and energy recovery was recommended but funding may be a barrier to implementation.^32^

Factors contributing to successful interventions include introduction of new technology (such as a well-performing scrubber control system), capacity-building and carbon tax policies.^32^^,^^34^^,^^36^ Barriers to successful implementation include unskilled operators, ineffective segregation and illegal removal of waste for recycling. Several policy interventions were suggested by the authors to deal with these potential barriers.^30^^,^^34^^,^^36^

##### Economic analysis

In a study from China, authors estimated that appropriate plastic recycling in the health-care system would lead to a cumulative economic benefit of about US$ 450 million in 2050.^34^ In another article, a cost–benefit analysis indicates that electricity generation from waste can cover a large portion of the fuel expenses of transportation and incineration of medical waste.^32^

##### Health and health equity

Reducing black carbon and sulfur emissions from incineration can reduce health risks, such as respiratory infections, low birth weight, premature deaths and asthma, in localities where incineration is happening nearby.^35^^,^^36^ Although waste burning is a relatively small contributor to black carbon globally, it is a substantial contributor to health-related illnesses in locations with high black carbon exposure such as in China, India, Nigeria and Republic of Korea.^48^

### Critical appraisal and risk of bias

Definitions of relevant methodological terms in the included studies were generally clear, but details on methods were missing in nine out of 22 (41%) articles. Fourteen studies (64%) reported on modelled outcomes, and eight (36%) reported on empirical outcomes. Some outcomes lacked transparency (missing data, time frames or units; six studies, 27%) and/or lack of confounding (eight studies, 36%). Seven articles (32%) did not clearly state assumptions, and 14 (64%) did not clearly state limitations. We did not note a conflict of interest partly because 12 articles (55%) did not include a conflict-of-interest statement. Funding sources included health ministry funds, government funds, national foundations and institutes, university grants, corporations,^23^ research councils and national programmes (Table 4). 

**Table 4:** Critical appraisal of studies included in the systematic review on greenhouse gas mitigation interventions for health-care systems

Country, reference	Definitions				Methods			Results			Confounding		Discussion	
	Clear definition of the objective or hypothesis?	Clear definition of intervention or exposure?	Clear definition of outcome?		Is/are the control(s) appropriate?	Methods applied consistently?		Data reported transparently?	Type of outcome measurement used?		Addressed in design or analysis?		Assumptions clearly stated?	Limitations clearly stated?
**Energy**														
Bangladesh^20^	Yes	Yes	Yes		Yes	Yes		Yes	Modelled		Yes		Yes	No
India^21^	Yes	Yes	Yes		Yes	Yes		Yes	Modelled		Yes		Yes	No
Malaysia^22^	Yes	Yes	Yes		Yes	Yes		No	Modelled		Yes		Yes	No
Philippines^23^	Yes	Yes	Yes		Yes	Yes		Yes	Modelled		Yes		Yes	Yes
India^24^	No	Yes	Yes		Yes	No		No	Empirical		No		No	No
Nigeria^25^	Yes	Yes	Yes		Yes	Yes		Yes	Modelled		Yes		Yes	No
Turkey^26^	Yes	Yes	Yes		Yes	NA		No	Modelled		Yes		No	No
India^27^	Yes	Yes	Yes		No	NA		Yes	Modelled		No		Yes	No
Brazil^28^	Yes	Yes	Yes		Yes	Yes		Yes	Modelled		Yes		Yes	No
India^29^	Yes	Yes	Yes		Yes	Yes		Yes	Modelled		Yes		Yes	No
**Waste**														
Pakistan^30^	Yes	Yes	Yes		Yes	Yes		No	Empirical		Yes		Yes	Yes
Türkiye^31^	Yes	Yes	Yes		Yes	Yes		Yes	Modelled		Yes		Yes	No
Pakistan^32^	Yes	Yes	Yes		Yes	Yes		Yes	Empirical		No		No	Yes
Malaysia^33^	Yes	Yes	Yes		Yes	NA		Yes	Empirical		No		No	Yes
China^34^	Yes	Yes	No		Yes	NA		Yes	Modelled		Yes		No	Yes
Haiti^35^	Yes	Yes	Yes		Yes	Yes		Yes	Empirical		No		Yes	No
Egypt^36^	No	No	Yes		Yes	Yes		Yes	Empirical		No		Yes	No
China^37^	Yes	Yes	Yes		Yes	Yes		No	Modelled		Yes		Yes	Yes
**Others**														
Malaysia^38^	Yes	Yes	Yes		No	NA		Yes	Modelled		No		No	No
India^39^	Yes	Yes	Yes		Yes	Yes		Yes	Empirical		Yes		No	Yes
China^40^	Yes	Yes	Yes		Yes	Yes		No	Modelled		Yes		Yes	No
India^41^	Yes	Yes	Yes		No	No		Yes	Empirical		No		Yes	Yes

NA: not applicable.

As no protocols were published in advance, we could not compare and identify selective reporting for any of the articles. None of the articles self-reported potential meta-biases.

#### Confidence in cumulative evidence

We evaluated confidence in the available evidence regarding the effect size of greenhouse gas emission reductions using the GRADE certainty assessment (Table 5), which is described in detail in the online repository.^15^ Across all 10 articles on energy, outcomes were assessed, as they spanned a variety of hybrid energy systems that included renewable energy resources. Regarding waste, we assessed four separate outcomes based on the different interventions described in the articles. The four remaining articles were assessed as separate outcomes in the text.

**Table 5:** Certainty of evidence for interventions to mitigate greenhouse gases for health-care systems, low- and middle-income countries

Outcome	Impact	No. of studies	Certainty of evidence^a^
Greenhouse gas mitigation through hybrid energy systems	A variety of hybrid energy systems, including renewable energy sources adjusted to contexts, reported reductions in carbon dioxide emissions ranging from 25% to a theoretical 233%	10 observational studies	Low
Greenhouse gas mitigation of health-care system waste through waste management systems with composting or recycling	Relative emission reductions are reported ranging between 46%–114% in systems that include waste segregation, composting, and material recycling while considering efficient low-emission transportation options	Four observational studies	Low
Greenhouse gas mitigation of health-care system waste through incineration and autoclave process efficiency	Relative emission reductions in waste management systems are reported to take place through centralizing the autoclave (reduces electricity needed), considering efficient transportation, and ensuring incinerators are up-to-date with a clear process and well-trained operator	Two observational studies	Very low^b^
Greenhouse gas mitigation of health-care system waste through replacing plastic sharps containers by cardboard sharps containers	Using cardboard sharps containers instead of plastic sharps containers led to a reported 62% reduction in black carbon emissions	One observational study	Very low^b^
Greenhouse gas mitigation of health-care system waste through microwave sterilization and landfilling	Urban medical waste management through microwave sterilization with landfill medical waste disposal technology reduces relative emissions as compared to rotary kiln incineration (68%), pyrolysis incineration (28%), plasma melting (80%) and steam sterilization with landfill (18%)	One observational study	Low
Greenhouse gas mitigation of health-care facility heating and cooling through heat exchangers	An eight-row heat pipe heat exchanger system added to one hospital ward was assessed to reduce carbon dioxide emissions compared to the regular air conditioning system by 147%, because of heat generation	One observational study	Low
Greenhouse gas mitigation of anaesthetic gases through induction dose only sevoflurane	Induction dose only sevoflurane during paediatric eye examination for children aged 1–5 years at one hospital reduces 22% of emissions compared to standard low-flow sevoflurane	One RCT	High
Greenhouse gas mitigation of a hospital building through lobby design	In this cold-climate region, a lobby with two exterior walls, south-oriented at the same height as the rest of the hospital, emits the least with a relative reduction of 0.014–0.074 kg CO_2_/m^2^ depending on the comparison design	One observational study	Very low^c^
Greenhouse gas mitigation of operations and logistics of cataract surgery	Multiuse pharmaceuticals, reusing surgical supplies, a short surgical duration and quick turnaround time resulted in a relative reduction of emissions of 95% as compared to the same surgery in the United Kingdom	One observational study	Very low
Climate adaptation from mitigation interventions	A solar photovoltaic panel energy system with and without grid-connection for a rural health-care facility in the Philippines may contribute to the resilience of a health-care facility to short-term disasters and events and as longer-term climate changes occur	One observational study	Very low^d^

CO_2_: carbon dioxide; RCT: randomized controlled trial.

^a^ We used the Grading of Recommendations Assessment, Development, and Evaluation approach.

^b^ Results (partially) based on visual observation of pollution.

^c^ Outcomes in electricity generated in carbon dioxide equivalent using national emission factors.

^d^ Adaptation was a consideration in the article and not measured.

## Discussion

Here we provide an overview of peer-reviewed evidence on greenhouse gas mitigation interventions for health-care systems in low- and middle-income countries. The eligible studies show reductions in greenhouse gas emissions, cost savings as well as potential positive health effects. Because the overall health sectoral emissions contribute to about 5% of global greenhouse gas emission, successful mitigation efforts need to be urgently scaled up to affect overall emissions. For example, in 2015, Chinese health-care systems emitted an estimated 302 megatonnes (Mt) of carbon dioxide, while the Kenyan and Malaysian systems emitted an estimated 2 Mt and 6 Mt of carbon dioxide, respectively.^2^ In our identified studies, the maximum reductions were approximately 0.9 Mt of carbon dioxide equivalent annually for a sustainable waste approach in China; and 0.02 Mt of carbon dioxide equivalent for a hybrid polygeneration energy system in a Brazilian hospital.^28^^,^^34^ However, due to the limited identified records and inconsistent methods, the overall quality of evidence is low and supports the conclusion that rigorous research, publication and dissemination is needed.

Fully renewable energy with battery storage, or hybrid energy systems including renewable and conventional sources provide a reliable and sustainable source of electricity, especially in areas with intermittent or unreliable grid electricity supply; and require decision-makers interested in implementing renewable energy systems to consider local conditions, such as energy prices, solar and wind parameters, and temperature to optimize performance and sustainability. A primary barrier to implementation is the high initial cost to purchase, install and maintain such systems or interventions. Irrespective of these barriers, we identified seven articles that reported positive returns, suggesting that the long-term benefits of implementing renewable energy systems outweigh the initial costs of implementation. Adequate funding is therefore crucial to support the initial setup of these mitigation interventions. 

Our results highlight actions such as waste segregation, composting and material recycling as means to reduce greenhouse gas emissions, which is consistent with evidence from other sectors and high-income country settings.^49^^,^^50^ Waste-to-energy technologies such as incineration, autoclaving and microwave sterilization could contribute more to greenhouse gas emission reductions than plasma melting or landfilling. We recommend that health-care facilities prioritize waste reduction, segregation and recycling, and address identified barriers through capacity-building and incentives before considering waste-to-energy technologies. However, identifying potential unintended negative consequences for the local community from waste produced by health-care facilities is essential, including pollution from incineration, when designing waste-management policies. Context-specific strategies to mitigate some of these effects need to be developed that are also sensitive to local socioeconomic and environmental conditions. Limited information on costs and potential benefits of waste management interventions in this systematic review underscores the need for further economic analysis.

There is evidence to suggest that building design optimization and improved surgical processes can lead to reductions in greenhouse gas emissions; however, there is a dearth of data on the implementation, costing and health impacts of these interventions.^38^^–^^41^ Although we have reviewed several promising interventions to reduce greenhouse gas emissions in health-care settings, there are gaps in our current knowledge of the implementation and sustainability of mitigation interventions and their potential scalability. These gaps restrict our understanding of the effects on overall sectoral emission reductions. Detailed information is lacking on the workforce required, the amount of implementation-related greenhouse gas emissions, and the time and resources needed for installation and deployment. Moreover, there is little information on other important issues such as long-term maintenance and upkeep.

This study has some limitations. First, the findings may not encompass all pertinent factors leading to successful implementation because of a lack of descriptive details. Second, the absence of consistent reporting methods in the literature restricts the comparability and generalizability of the results and impedes further in-depth analysis. Third, the GRADE approach is designed for single interventions, which creates challenges in the interpretation of systemic change. To overcome these limitations, further research is necessary to obtain more comprehensive evidence on the effectiveness, scalability and durability of mitigation interventions in health-care systems in low- and middle-income countries using standard approaches; for example by adapting guidelines for evaluation of complex interventions to the planetary health agenda.^51^^,^^52^

We found that the types of interventions reported in the literature are limited to a few areas that contribute to emissions, namely energy, waste, heating and cooling, operations and logistics, building design and anaesthetic gases. We also noted a lack of reported interventions in other subject areas including equipment efficiency; inhalers; food; manufacturing and efficient use of pharmaceuticals and chemicals; production, reduction and circularity of medical supplies and devices; partnerships, purchasing and finance; information and communication technologies; telemedicine; community-based care; and supply-chain management.^8^ Further, interventions focusing on systemic efficiencies of delivery of high-quality care were not identified and improving the efficiency of health-care provision could provide another opportunity to reduce emissions (Box 3). 

There is a lack of data on how to consider context-specific adaptation and mitigation measures, particularly in low- and middle-income countries. Future research and interventions should consider a wider range of contexts, including low-income countries, all scopes of emissions and adaptation. While efforts are increasing to mitigate greenhouse gas emissions from health-care systems, such as through WHO's Alliance for Transformative Action on Climate Change and Health,^53^ it is essential to robustly monitor, evaluate, record and report outcomes in a standardized manner. An example of a tool that could support such efforts is the recently launched HealthcareLCA database, which contains assessments focused on the environmental impact of health care.^54^ In addition, reviewing grey literature such as reports from nongovernmental organizations, local organizations and community-based initiatives could provide valuable insights into the implementation and sustainability of interventions in low- and middle-income countries. Adding grey literature can complement findings from academic research and fill gaps in knowledge, particularly in resource-constrained settings where formal research may be limited. Such evidence will, however, require critical assessment because of the potential for methodological weaknesses and conflicts of interest leading to biased findings.

In conclusion, this review illustrates a wide range of interventions to mitigate greenhouse gas emissions in health-care systems in low and middle-income countries. We also highlight important gaps in the research-based knowledge. Further research, monitoring and evaluation are necessary to establish a robust evidence base and inform future policy decisions and interventions towards successful greenhouse gas mitigation and adaptation of health-care systems in the context of climate change.
